# Incorporation of Soil-Derived Covariates in Progeny Testing and Line Selection to Enhance Genomic Prediction Accuracy in Soybean Breeding

**DOI:** 10.3389/fgene.2022.905824

**Published:** 2022-09-08

**Authors:** Caio Canella Vieira, Reyna Persa, Pengyin Chen, Diego Jarquin

**Affiliations:** ^1^ Division of Plant Science and Technology, Fisher Delta Research, Extension and Education Center, University of Missouri, Portageville, MO, United States; ^2^ Agronomy Department, University of Florida, Gainesville, FL, United States

**Keywords:** genomic prediction/selection, genotype × environment G×E interaction, soil covariates, genetic gain, soybean breeding

## Abstract

The availability of high-dimensional molecular markers has allowed plant breeding programs to maximize their efficiency through the genomic prediction of a phenotype of interest. Yield is a complex quantitative trait whose expression is sensitive to environmental stimuli. In this research, we investigated the potential of incorporating soil texture information and its interaction with molecular markers *via* covariance structures for enhancing predictive ability across breeding scenarios. A total of 797 soybean lines derived from 367 unique bi-parental populations were genotyped using the Illumina BARCSoySNP6K and tested for yield during 5 years in Tiptonville silt loam, Sharkey clay, and Malden fine sand environments. Four statistical models were considered, including the GBLUP model (M1), the reaction norm model (M2) including the interaction between molecular markers and the environment (G×E), an extended version of M2 that also includes soil type (S), and the interaction between soil type and molecular markers (G×S) (M3), and a parsimonious version of M3 which discards the G×E term (M4). Four cross-validation scenarios simulating progeny testing and line selection of tested–untested genotypes (TG, UG) in observed–unobserved environments [OE, UE] were implemented (CV2 [TG, OE], CV1 [UG, OE], CV0 [TG, UE], and CV00 [UG, UE]). Across environments, the addition of G×S interaction in M3 decreased the amount of variability captured by the environment (−30.4%) and residual (−39.2%) terms as compared to M1. Within environments, the G×S term in M3 reduced the variability captured by the residual term by 60 and 30% when compared to M1 and M2, respectively. M3 outperformed all the other models in CV2 (0.577), CV1 (0.480), and CV0 (0.488). In addition to the Pearson correlation, other measures were considered to assess predictive ability and these showed that the addition of soil texture seems to structure/dissect the environmental term revealing its components that could enhance or hinder the predictability of a model, especially in the most complex prediction scenario (CV00). Hence, the availability of soil texture information before the growing season could be used to optimize the efficiency of a breeding program by allowing the reconsideration of field experimental design, allocation of resources, reduction of preliminary trials, and shortening of the breeding cycle.

## Introduction

Soybean [*Glycine max* (L.) Merr.] represents the largest and most concentrated segment of global agricultural trade (Gale et al., 2019). It is the crop that delivers the highest amount of protein per hectare and accounts for over 60% of total global oilseed production (United States Department of Agriculture, 2022). Worldwide, Brazil (37%, 139,000 MT), United States (32%, 120,700 MT), and Argentina (12%, 46,500 MT) account for over 80% of the soybean production (United States Department of Agriculture, 2022). Over the last two decades (2001/2002 to 2021/2022), soybean production has nearly doubled from 182,830 to 363,860 MT (United States Department of Agriculture, 2002; United States Department of Agriculture, 2022). The substantial increase in soybean production can be attributed to advances in agronomical practices (Specht et al., 1999; Mourtzinis et al., 2018), faster implementation of novel technologies in farming operations (Liu et al., 2008; Ainsworth et al., 2012; Vieira and Chen, 2021), and the development of improved soybean cultivars (Salado-Navarro et al., 1993; Voldeng et al., 1997; Specht et al., 1999; Specht and Williams, 2015; Vieira and Chen, 2021), of which the availability of high dimensional genomic (Song et al., 2013, 2020) and phenomic data (Moreira et al., 2019, 2020; Parmley et al., 2019; Zhou et al., 2022), as well as the integration of environmental covariates (ECs) through predictive analytics, have contributed to accelerated genetic gains (Jarquin et al., 2014a; Jarquin et al., 2014b; Persa et al., 2020; Widener et al., 2021).

Marker-assisted selection (MAS) has greatly contributed to the improvement and selection of soybean traits regulated by major effect genes, including biotic (Pham et al., 2013; Shi et al., 2015) and abiotic tolerance (Pathan et al., 2007; Wu et al., 2020), as well as seed composition–related traits (Pham et al., 2010; Patil et al., 2017). On the other hand, yield is a highly complex quantitative trait regulated by many genes with small effects, thus limited success has been reported in applying MAS (Concibido et al., 2003; Jarquin et al., 2014a). Bernardo (1994) was the first one who proposed the use of genomic variants (RLFPs) for predicting trait performance for selecting genotypes (genomic selection, GS), back then, the number of these covariates was limited/reduced. Later, Meuwissen et al. (2001) proposed a new methodology to deal with the curse of the dimensionality problem (*n* < *p*; *n* is the number of data points available for model fitting and *p* is the number of genomic variants) and it is considered a landmark in genomic research. The concept of GS revolves around using the information of all molecular markers—large and small effects—to develop prediction models for the phenotype of interest. The major advantage of GS relies on the ability to predict the yield of genotypes to allow the identification and selection of the most promising individuals earlier in the breeding pipeline, which not only reduce costs, time, and space but enhance the genetic gain by reducing the length of the breeding cycle, increasing selection intensity, as well as allowing the breeders to have a clear knowledge of the genetics of the materials early in the pipeline (Jarquin et al., 2014b; Crossa et al., 2017; Vieira and Chen, 2021; Wartha and Lorenz, 2021).

In soybean, the first application of GS was reported by Jarquin et al. (2014a). By using a standard G-BLUP model including only additive effects and an extended version of the G-BLUP model including additive-by-additive effects, a prediction accuracy of 0.64 for grain yield and roughly 41% of the phenotypic variance explained by the genotypic component were reported using 301 lines of the University of Nebraska soybean breeding program. Usually, different response patterns in a set of genotypes are observed when these are tested under different environmental conditions complicating the selection of the most promising candidates (Crossa et al., 2004). The presence of these changes in the response pattern of the ranking of the genotypes is also known as the genotype-by-environment interaction effect. To allow the consideration of this interaction effect in prediction models using weather data, Jarquin et al. (2014b) proposed a reaction norm model that allows the incorporation of the main and interaction effects of both high-dimensional molecular markers and EC through covariance structures using data from wheat cultivars tested in 340 environments. In the cross-validation scenario that considers the prediction of the performance of genotypes that have never been evaluated in field trials (CV1), in comparison with the conventional main effect genomic selection model, the reaction norm model enhanced prediction accuracy by 35%, whereas in the cross-validation scenario where all genotypes had at least one field evaluation available (CV2), a 17% increase in predictive ability was observed (Jarquin et al., 2014b). Using the soybean nested association panel (SoyNAM), Xavier et al. (2016) investigated the impacts of training population size, genotyping density, and 14 prediction models on the accuracy of genomic prediction. These authors showed that the training population size was the most impactful factor in the accuracy improvement. Ma et al. (2016) used ridge regression best linear unbiased prediction (rrBLUP) (Endelman, 2011) with fivefold cross-validation to explore strategies of marker preselection. The prediction accuracy based on markers selected with a haplotype block analysis–based approach increased by approximately 4% compared with random or equidistant marker sampling. Stewart-Brown et al. (2019) investigated the effects of two relatedness strategies among genotypes in overall prediction accuracies and found both methods returned similar accuracies. The first method was based on each bi-parental population and utilized a training set of full-sibs of the validation set. The second method utilized a training set of all remaining breeding lines except for full-sibs of the validation set to predict across populations. Persa et al. (2020) expanded the reaction norm model proposed by Jarquin et al. (2014b) by incorporating the interaction between genotypes’ families and the environment under the premise that the differential responses of families to environmental stimuli could be used for enhancing the selection process in target environments. The most comprehensive model improved the predictive ability by 41% (CV1) and 49% (CV2) compared to the standard GBLUP, and roughly 17% as compared to the conventional reaction norm model. Widener et al. (2021) included three EC (mean minimum daily temperature, mean maximum daily temperature, and mean daily precipitation) interactions with molecular markers into the reaction norm model and no substantial increase in prediction accuracy was observed and resulted in more often negative predictions although these authors were only interested in assessing strategies to selecting sets of environments for model training. These authors found that in predicting the most dissimilar environment (based on phenotypes and environmental covariates) a reduced set of environments is adequate to optimize predictive ability while for the most similar environment, as the number of environments in the training set increased the predictive ability was improved too.

The covariance structure proposed in the reaction norm allows the borrowing of information between genotypes based on environmental and genomic similarities. For instance, in Jarquin et al. (2014b), the covariance matrices describing the similarities between environmental conditions and genetic information permit the borrowing of information between environments and molecular markers, respectively. The cross-validation scenario where untested genotypes are being predicted in untested environments (CV00) is often the challenge faced in the early stages of a breeding pipeline also known as the progeny stage. In this situation, the environmental conditions in upcoming growing seasons are often unpredictable and distinct from what was used in the model’s training dataset limiting the main advantage of the approach based on conventional covariance structures only. Soil-related information such as soil texture is generally constant across years and readily available before the growing season. Consequently, leveraging the information of soil texture as the main effect as well as its interaction with molecular markers could potentially increase predictive ability, particularly in scenarios considering untested genotypes in untested environments. Therefore, the objective of this study was to investigate the potential of including soil-derived covariates in the reaction norm model to enhance the predictive ability under common plant breeding scenarios, including the prediction of untested genotypes in untested environments (progeny testing) as well as multiple combinations of tested genotypes in tested and untested environment simulating line selection. A set of 797 advanced soybean breeding lines derived from unique 367 bi-parental populations was used in this study. Lines were evaluated for grain yield between 2017 and 2021 and genotyped using the Illumina Infinium BARCSoySNP6K BeadChip.

## Materials and Methods

### Plant Materials and Field Trials

A set of 797 advanced soybean breeding lines derived from 367 unique bi-parental populations developed by the University of Missouri–Fisher Delta Research, Extension, and Education Center (MU-FDREEC), soybean breeding program was used in this study. The lines comprised 5 years (2017–2021) of internal advanced yield trials at the MU-FDREEC. Five seeds of each line were germinated in paper pouches for 3–4 days at room temperature and seedlings were transplanted into micropots filled with sterilized sandy loam soil. Genomic DNA was extracted from lyophilized young trifoliate leaf tissue (V3) (Fehr et al., 1971) using the Qiagen DNeasy Plant 96 kit (QIAGEN, Valencia, CA, United States) and respective protocol. DNA concentration was quantified using a spectrophotometer (NanoDrop Technologies Inc., Centerville, DE, United States) and normalized at 50 ng/μl. DNA samples were genotyped in the USDA-ARS Soybean Genomics and Improvement Laboratory using the Illumina Infinium BARCSoySNP6K BeadChip (Song et al., 2020). The single nucleotide polymorphism (SNP) alleles were called using the Illumina Genome Studio Genotyping Module (Illumina, Inc., San Diego, CA, United States).

Field trials were conducted for 5 years (2017–2021) at the Lee Farm in Portageville, MO (36°23′44.2″N latitude and 89°36′52.3″W longitude) and the Rhodes Farm in Clarkton, MO (36°29′14.8″N latitude and 89°57′39.0″W longitude) using a three-replicate randomized complete block design. At the Lee Farm, trials were conducted each year in four environments consisting of two Tiptonville silt loam and two Sharkey clays. Tiptonville silt loam consists of very deep, nearly level, moderately well-drained soils formed in silty alluvium (United States Department of Agriculture, 2018a), whereas Sharkey clay is very deep, poorly drained, and very slowly permeable in soils that is formed in clayey alluvium (United States Department of Agriculture, 2013). At the Rhodes farm, trials were conducted in one Malden fine sand environment each year. This consists of very deep, excessively drained soils formed in sandy alluvium (United States Department of Agriculture, 2018b). Each plot consisted of four rows 3.66 m long spaced 0.76 m apart. The two center rows of each plot were harvested with a plot combined for seed yield adjusted to 13% seed moisture.

### Statistical Models

For assessing the effects of the soil type–derived covariates and their interactions with environmental factors in genomic prediction, four models were considered.

#### M1: E+L+G

This model allows the inclusion of the main effect of the molecular markers *via* covariance structures. Suppose that the genomic effect $g_{i}$ of the *i*th line can be characterized by a linear combination between *p* molecular markers $x_{m}$ (m=1,2, …,p) and their corresponding effects $b_{m}$ such that $g_{i}=\overset{p}{\underset{m=1}{\sum }}x_{m}b_{m}$ , with $b_{m}∼N\left(0,\sigma_{b}^{2}\right)$ . If we include all the genomic effects into a single vector, we have g=Xb . From results of the multivariate normal density, the vector of genomic effects $g=\left\{g_{i}\right\}∼N\left(0,G\sigma_{g}^{2}\right)$ with $G=\frac{XX^{\prime }}{p}$ , and $\sigma_{g}^{2}=p\sigma_{b}^{2}$ as the corresponding variance component. In this way, the linear predictor becomes.

$$y_{ij}=\mu +E_{j}+L_{i}+g_{i}+\varepsilon_{ij}$$ (1)

where the yield response $y_{ij}$ of the *i*th genotype observed at the *j*th environment can be modeled as the sum of a mean effect µ common to all genotypes across environments, a random effect of the *i*th line $L_{i}$ following an independent and identically distributed (IID) normal density centered on zero and variance $\sigma_{L}^{2}$ such that $L_{i}∼N\left(0,\sigma_{L}^{2}\right)$ , a random environmental effect of the *j*th environment $E_{j}$ following IID normal densities centered on zero and variance $\sigma_{E}^{2}$ such that $E_{j}∼N\left(0,\sigma_{E}^{2}\right)$ , and a random effect $\epsilon_{ij}$ addressing the unexplained variability by these model terms such that $\epsilon_{ij}∼N\left(0,\sigma_{\epsilon }^{2}\right)$ .

#### M2: E+L+G+G×E

To consider the effect of the environmental stimuli on the genomic responses, Jarquin et al. (2014b) proposed the reaction norm model. Briefly, this model indirectly allows the inclusion of the interaction between each molecular marker and each environment or environmental covariate in prediction models *via* covariance structures. Consider $gE_{ij}$ as the random effect explaining the genomic interaction between the *i*th genotype and the *j*th environment such that the vector of interaction effects $gE=\left\{gE_{ij}\right\}∼N\left(0,Z_{E}Z_{E}^{\prime }\#Z_{L}GZ_{L}^{\prime }\sigma_{gE}^{2}\right)$ , where $Z_{L}$ and $Z_{E}$ are the incidence matrices that connect phenotypes with genotypes and environments, respectively, $\sigma_{\text{gE}}^{2}$ is the corresponding variance component, and “#” represents the Hamadard product (cell-by-cell product) between two matrices of the same dimensions. Adding this model term to M1 results in the following linear predictor:

$$y_{ij}=\mu +E_{j}+L_{i}+g_{i}+gE_{ij}+\varepsilon_{ij}$$ (2)

This model has shown significant improvements in predictive ability compared with the conventional GS model (M1) when predicting the yield of genotypes in already observed environments. However, in more challenging scenarios like those where no phenotypic records from the target environment are available for any genotype, the advantage becomes less pronounced likely due to the environmental stimuli not being properly accounted for. Also, predicting future environments poses an extra challenge since it is not feasible to forecast the expected weather conditions in a precise manner limiting the usefulness of M2 in these cases.

#### M3: E+L+S+G+G×E+G×S

An important component of the environmental stimuli that genotypes are exposed to is the multiple soil conditions, of which soil structures are factors that can be easily obtained in advance during the planning stage of the experiments. The current model attempts to leverage the information on the soil structure in the prediction context. Consider $S_{k}$ as the random effect that represents the soil type where the soybean cultivars were planted (*k* = 1, 2,…, *K*). Furthermore, if we assume these effects as IID outcomes from a normal distribution centered on zero and with a common variance $\sigma_{S}^{2}$ we have $S_{k}∼\text{N}\left(0,\sigma_{S}^{2}\;\right)$ . This model term allows the inclusion of the main effect of the soil type in the prediction model. In principle, it is assumed that the effect of soil type is the same for all genotypes planted in a given experiment. Thus, this model term will not help to improve the predictive ability because their effects are common to all genotypes within the same experiment. For this reason, we also considered the interaction between the molecular markers and the soil type to permitting specific responses within environments also allows the borrowing of information between genotypes planted at different soil types. For this, we used the same principles as in M2 such that $gS_{ik}$ represents the interaction effect of the *i*th genotyped at the *k*th soil type. If we include these interaction effects in a vector we have $gS=\left\{gS_{ik}\right\}∼N\left(0,Z_{L}GZ_{L}^{\prime }\#Z_{S}Z_{S}^{\prime }\sigma_{gS}^{2}\right)$ , where $Z_{S}$ is the incidence matrix that connects phenotypes with the soil type where the genotypes are observed, and $\sigma_{gS}^{2}$ represents the associated variance component. Combining this model term with M3, we have the resulting linear predictor.

$$y_{ij}=\mu +E_{j}+S_{k}+L_{i}+g_{i}+gE_{ij}+gS_{ik}+\varepsilon_{ij}$$ (3)

where all of the remaining terms remain as previously defined.

#### M4: E+L+S+G+G×S

Finally, a fourth model (M4) results from dropping the G×E term from M3. It is an attempt to have an intermediate implementation between models M2 and M3. The resulting model is as follows:

$$y_{ij}=\mu +E_{j}+S_{k}+L_{i}+g_{i}+gS_{ik}+\varepsilon_{ij}$$ (4)

where all of the remaining terms remain as previously defined.

### Cross-Validation Schemes

In this study, four cross-validation schemes that simulate realistic prediction scenarios of interest for breeders for screening, selecting, and advancing genotypes through the breeding pipeline were implemented. The goal of considering these four prediction scenarios is to evaluate if in any of these the integration of soil-derived covariates accomplishes significant improvements in predictive ability. Persa et al. (2020) provide a comprehensive review of these four cross-validation scenarios and an extension to balancing the sample sizes in training and testing sets across cross-validation schemes.

The first prediction scenario is called CV2 (tested genotypes in observed environments), and it refers to the problem of predicting already tested genotypes in already observed environments. The main purpose of this scheme is to assess the predictability of partial field trials. Few genotypes have already been observed in some environments but not in others and the interest is to predict their performance in those environments where these genotypes were not observed. In this study, a fivefold cross-validation was considered such that around 20% of the phenotypic values were assigned to the testing set and the remaining 80% (or four folds) to the training set which is used for model calibration. The model evaluation was conducted by predicting each fold (one at a time) using the remaining four folds for calibration, and this procedure was repeated until all the five folds were completed. This previous procedure was repeated 10 times.

The second prediction scenario is CV1 (untested genotypes in observed environments), and it refers to the problem of predicting untested genotypes in already observed environments where other genotypes were already tested. This prediction scenario mimics the problem of predicting (novel or newly developed) genotypes that were not observed in any of the environments; however, in these environments there is available phenotypic information for other genotypes. Even though the phenotypic information for these target genotypes of interest is not available, it is possible to borrow information from other genotypes *via* genomic data to allow the prediction of the unobserved genotypes. Also, a fivefold cross-validation was considered. In this CV, genotypes were assigned to folds instead of phenotypes such that all phenotypic records from the same genotype are encountered in the same fold. Under this scenario, around 20% of the genotypes were used as validation or testing set and the rest (∼80% of the genotypes) were considered for the model’s calibration. Similarly, to CV2, each fold was predicted (one at a time) using the remaining four folds and this procedure was repeated 10 times.

The CV0 (tested genotypes in unobserved environments) cross-validation scheme considers the scenario of predicting the performance of already observed genotypes in other environments and the interest is to predict their performance in an unobserved/novel environment. Under this scheme, the genotypes’ mean performance is predicted in a hypothetical unobserved environment. The training set includes phenotypic records from all the genotypes in these already observed environments. The validation is conducted by predicting the performance of all the lines in one unobserved environment (one at a time) using the information of the remaining environments (training set). These steps are repeated for every environment.

CV00 (untested genotypes in unobserved environments) is perhaps the most interesting cross-validation scenario for breeders but also it is the most challenging. It considers the prediction of novel genotypes that have not been tested in any environment yet, and breeders are interested in their performance in an unobserved/novel environment. The strategy for estimating untested genotypes in new environments consists of removing all the phenotypic information from the target environment as well as all the phenotypic information from the training set but corresponding to only to those genotypes in the testing set (unobserved environment).

### Model Assessment

The predictive ability of the different models for the different cross-validation schemes was calculated as the within-environment correlation between the predicted and observed values. These correlations provide an assessment of the model’s predictive ability at the environment level which may vary substantially across environments due to a large number of unaccounted environmental conditions and sample sizes of the environments.

A general assessment across environments predictability is obtained by computing the weighted average correlation to account for uncertainty and the sample size of the environments as proposed by Tiezzi et al. (2017).

$$r_{w}=\;\frac{\sum_{j=1}^{J}\frac{r_{j}}{V\left(r_{j}\right)}}{\sum_{j=1}^{J}\frac{1}{V\left(r_{j}\right)}}$$

where $V\left(r_{j}\right)=\frac{1-r_{j}^{2}}{n_{j}-2}$ , $r_{j}$ represents the Pearson correlation between predicted and observed records at the $j^{th}\left(w=1,\;\ldots ,\;50\right)$ environment; $V\left(r_{j}\right)$ and $n_{j}$ corresponds to the sampling variance and number observations, respectively.

### Variance Components

In general, the addition of model terms would result in a change in the predictive ability. To assess the importance/contribution of these terms, a full data analysis (i.e., non-missing values) was conducted to compute the variance components and examine the relative contribution of the different model components for each model. For this, the proportion of explained variability from each model term *z* is calculated as the ratio of the associated variance component to the sum of all *t* variance components (*z* = 1, 2,..,*t*) in the model multiplied by 100

$$\left(\frac{\sigma_{z}^{2}}{\sum_{z=1}^{t}\sigma_{z}^{2}}\;\times \;100\right)$$

## Results

### Variance Components

The relative amount of phenotypic variability (percentage) explained by the different model terms (across and within environments) for the four models (M1–M4) is provided in Table 1. Across environments, in M1; the environment component (E) captured the largest amount of phenotypic variability (65.7%) while the lines (L) and the main effect of the markers (G) explained 1.2 and 7.3% respectively, and the remaining non-explained variability addressed by the error term (R) was 25.8%. The addition of model terms significantly reduced the amount of variability captured by the E and R terms. Under the most complex model (M3), the corresponding values were reduced by 30.4% (45.7%) and 39.2% (15.7%), respectively. Concerning the percentage of within environment variability (after discarding the E term), the residual term R captured 75.2% with M1 while with M3, it was reduced by almost threefold to 28.9%. The interaction between molecular markers and environments (G×E) and between molecular markers and soil type (G×S) explained 20 and 17.5% of the phenotypic variability, respectively. These results highlight the importance of considering the interaction between molecular markers and environmental descriptors (environments and soil type) with the potential for improving predictive ability.

**TABLE 1:** Percentage of phenotypic variability explained by the different model components across and within environments for the four models (M1–M4).

Model	% Of across environment variability								% Of within environment variability					
	E^a^	L	S	G	G×E	G×S	R		L	S	G	G×E	G×S	R
M1: E+L+G	65.7	1.2		7.3			25.8		3.6		21.3			75.2
M2: E+L+G+G×E	65.7	1.6		6.0	12.7		14.0		4.5		17.5	37.1		40.9
M3: E+L+S+G+G×E+G×S	45.7	2.2	12.5	3.5	10.9	9.5	15.7		4.0	23.1	6.5	20.0	17.5	28.9
M4: E+L+S+G+G×S	64.0	1.5	0.0	4.2		9.5	20.8		4.2		11.6		26.4	57.8

a The letters E, L, S, and G denote the mean effects of environments, genotypes, soil type, and molecular markers, respectively, whereas G×E and G×S reflect the interaction of each molecular marker with environments and soil type, respectively. The residual variance is denoted by R.

### Predictive Ability

A very quick assessment of the ability of the different models for performing predictions can be achieved by revising the within environment mean average correlation between predicted and observed values. Table 2 displays the mean average correlations for the four cross-validation schemes (CV2, CV1, CV0, and CV00) and to the four prediction models (M1–M4), and the results of the best model are highlighted in boldface by columns. Under the incomplete field trial scenario (CV2), the best model was M3 (0.577) which improved the conventional genomic selection model (M1) by 25.1% and was approximately 4% superior to the reaction norm model including G×E (M2). For the scenario of predicting newly developed lines in observed environments (CV1), models M2 and M3 performed similarly (∼0.48), outperforming M1 by 34%. When predicting the yield of already tested genotypes in novel environments (CV0), the inclusion of G×E and G×S did not provide substantial improvement in overall accuracy as observed in the other cross-validation scenarios. In this case, the best model was M3 (0.488) which slightly outperformed M1 (0.461), M2 (0.459), and M4 (0.484). Thus, an improvement of 6% in the predictive ability was observed in M3 as compared to M1. In the most challenging and interesting prediction scenario consisting of predicting new genotypes in novel environments (CV00), the main effect model M1 returned the highest average correlation (0.240), followed by M4 (0.231), M3 (0.227), and M2 (0.192). In general, when considering only the mean average correlation as the unique criteria for selecting the best prediction model, M3 outperformed the other models in CV2, CV1, and CV0, while under CV00 the conventional main effect model M1 yielded the highest predictive accuracy.

**TABLE 2:** Weighted mean average correlation across environments for four cross-validation schemes and four models.

Model^a^	CV2^b^	CV1	CV0	CV00
M1: E+L+G	0.461	0.359	0.461	**0.240** ^c^
M2: E+L+G+G×E	0.558	**0.480**	0.459	0.192
M3: E+L+S+G+G×E+G×S	**0.577**	**0.480**	**0.488**	0.227
M4: E+L+S+G+G×S	0.515	0.405	0.484	0.231

a E, L, S, and G constitute the main effect of the environments, genotypes, soil type, and molecular markers; and G×E and G×S evoke the interaction between each molecular marker with environments and soil type, respectively.

b CV2 considers the case of predicting incomplete field trials (i.e., some genotypes tested in some environments but not others), whereas CV1 assessed the accuracy of predicting newly developed genotypes. CV0 represents plant performance in novel environments of previously studied genotypes. CV00 assesses new genotypes in novel environments. For CV2 and CV1, 10 replicates of fivefold cross-validation were considered while for CV0 and CV00 the leave one environment out scheme was implemented.

c Bolded numbers indicate the best model performance for each cross-validation scheme.

### Within Environment Predictive Ability as a Function of the Sample Size

Supplementary Figures S1, S2 in the Supplemental Section display the within-environment average correlation (*y*-axis) between predicted and observed values (10 replicates of fivefold cross-validation) as a function of the sample size of the environments (*x*-axis) under CV2 and CV1 prediction scenarios for the four models (M1–M4). For CV0 and CV00, since these do not involve a randomization process because each environment is left out at a time, the correlation between predicted and observed values are computed only once within environments and their corresponding results are displayed in Supplementary Figures S3, S4, respectively. For the four cross-validation schemes, the correlations for each model-environment are provided in Supplemental Section in Supplementary Tables S1–S4.

Under the CV2 scenario, in Supplementary Figure S1 (Supplementary Table S1); we observed that as the number of genotypes in the target environment increased the mean average correlation also increased. Negative correlations were observed with the M1 (panel A) model in 11 of the 50 environments, while these negative values were observed with the M2, M3, and M4 models in only six, four, and four environments, respectively. For the CV1 scenario, a similar trend to the previous prediction scheme was observed. The main effect model M1 returned negative values in eight environments, M2 returned negative values in only five environments, M3 returned the lowest number of environments with adverse outcomes (3), and the intermediate model M4 resulted in five environments with negative correlations (Supplementary Figure S2 and Supplementary Table S2). In the CV0 scheme, the model M1 returned nine out of the 50 environments with negative correlations while with M2 10 out of the 50 environments resulted in negative correlations (Supplementary Figure S3 and Supplementary Table S3). The interaction models that consider the soil type (M3 and M4) resulted in only five environments with negative results. Regarding the most complex prediction scenario CV00, the main effect model M1 returned negative results in nine out of the 50 environments, M2 resulted in 10 environments with adverse outcomes while M3 and M4 returned only six and five environments with negative correlations, respectively (Supplementary Figure S4 and Supplementary Table S4).

### Predictive Ability of Genotypes in Environments

Another way to assess model predictive ability was introduced by Jarquin et al. (2014b). These authors superimposed a grid on the scatter plot between predicted and observed values with the grid's vertical and horizontal lines represent the empirical percentiles (20, 50, and 80%) of the predicted and observed values, respectively. Also, within each rectangle of the grid, the proportion of genotypes at each category in the *y*-axis (observed values) conditional on the groups/categories defined by the *x*-axis (predicted values) is displayed. Supplementary Figures S5–S8 in Supplemental Section contain the corresponding conditional plots for the four cross-validations and the four models.

For CV2, among the top 20% (i.e., to the right from the vertical line in the 80% mark on the *x*-axis) of the predicted genotypes in environments with model M1 (top right panel A), 68% of these showed an observed performance among the top 20% (i.e., above the 80% of the horizontal line in the *y*-axis) phenotypes in fields (Supplementary Figure S5). On the other hand, out of the bottom 20% (i.e., to the left from the vertical line in the 20% mark on the *x*-axis) of the genotypes predicted to have the lowest performance in fields, 71% were among the observed genotypes with the poorest performance. In addition, a linear regression between the predicted and observed values was performed, as well the mean squared error (MSE) and the weighted average correlation across environments (Cor) were added to the plot. An R-square (*R* ^2^) of 0.66 resulted from regressing the observed values on the predicted values, MSE = 94.1 and a Cor = 0.461.

Using the M2 model, 71% of the genotypes projected to have superior performance in fields (i.e., among the top 20%) were classified in the right category while 74% of those predicted with the poorest performance were among the phenotypes with the lowest performance. The resulting *R* ^2^ was 0.72 for a MSE = 77.6 and a Cor = 0.558. The most complex model M3, returned classification successes of the top and the worse genotypes in fields of 71 and 76%, respectively, for an *R* ^2^ = 0.73, MSE = 75.7, and a Cor = 0.577. For the intermediate model M4, the corresponding classification successes were 69% (top 20%) and 74% (bottom 20%) with *R* ^2^ = 0.69, MSE = 86.2, and a Cor = 0.515.

For the CV1 cross-validation scheme, M1 (Supplementary Figure S6) returned a classification success of 67% for the top 20% of the genotypes in fields and 70% for those with the poorest performance, with an *R* ^2^ = 0.64, MSE = 100.8, and a Cor = 0.359. With M2 the corresponding classification successes were 71 and 73%, with an *R* ^2^ = 0.7, MSE = 84.7, and Cor = 0.48. Similar values to those obtained with M2 were obtained with M3 for all the mentioned criteria. Finally, with M4 a slight reduction in the classification success was observed for the top 20% (67%) and the lowest 20% (71%) as compared to M2 and M3, with an *R* ^2^ = 0.66, MSE = 95.1, and Cor = 0.405.

When predicting already tested genotypes in untested environments (CV0), M1 returned a low classification success of the top and bottom 20% genotypes (25 and 29%, respectively), with an *R* ^2^ = 0.03, MSE = 291.8, and a Cor = 0.461. There, M2 and M3 returned similar results to those from M1 with a slight decrease in the MSE and a slight improvement of the weighted average correlation with M3 (0.488) (Supplementary Figure S7). The most promising model in this scenario was M4 which returned a classification success of the top and bottom 20% of 34 and 36%, respectively. It also returned the highest *R* ^2^ (0.11) and the smallest MSE (251.9) among all models leveraging the advantage of including soil type in interaction with molecular markers in the prediction models.

For the most complex prediction scenario CV00, the classification success rate, *R* ^2^, and Cor values were reduced across all models while the MSE increased simultaneously. M1 returned a classification success rate of the top and bottom 20% performing lines of 17 and 25%, respectively, with an *R* ^2^ = 0, MSE = 305, and a Cor = 0.24. In M2, M3, and M4, the average weighted correlation was reduced to 0.192, 0.227, and 0.231, respectively (Supplementary Figure S8). However, the classification success of the top and bottom performing lines was improved with M3, especially on the ability to detect the top 20% genotypes. For this model, the classification success was 29% for identifying the top 20% genotypes while it was 26% for screening out the worst-performing genotypes.

### Overall Performance of Genotypes

**FIGURE 1:**
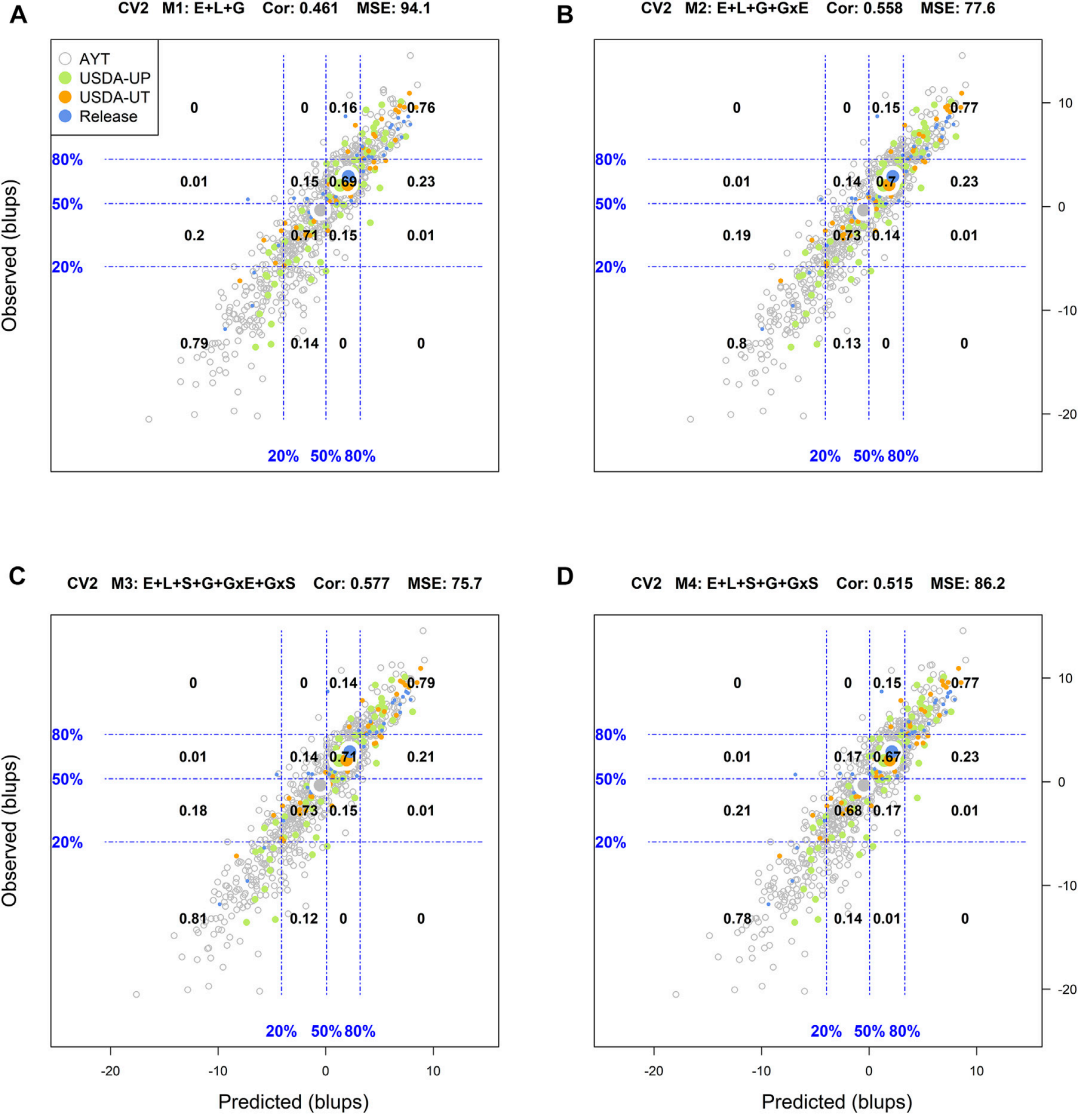
Genotypic means (BLUP-centered on zero within environments) of observed versus predicted cross-validation predictions of four models (M1–M4) under the cross-validation scheme CV2, which mimics the incomplete field trial prediction scenario (predicting tested genotypes in observed environments). Models and terms are described in detail in the *Materials and Methods* section (Eqs 1–4). Horizontal and vertical dashed lines indicate the 20, 50, and 80% empirical percentiles corresponding to the genotypic means of observed and predicted values; the numbers inside the grid provide the conditional proportions observed on the *y*-axis for the different percentiles on the *x*-axis (e.g., out of the top 20% of the predicted values with M3 (panel C), 79% [top right] of these correspond to genotypes that showed a performance among the 20% across fields).

**FIGURE 2:**
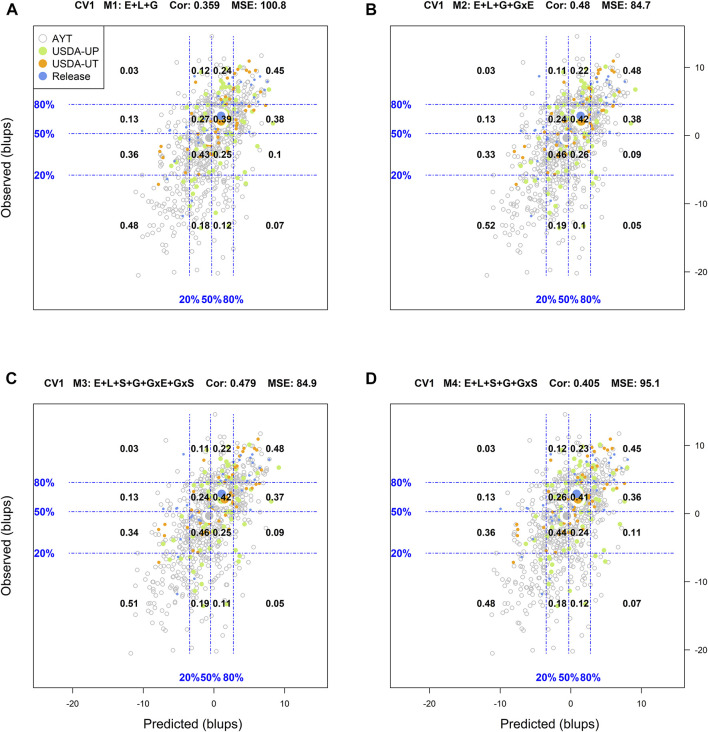
Genotypic means (BLUP-centered on zero within environments) of observed versus predicted cross-validation predictions of four models (M1–M4) under the cross-validation scheme CV1, which mimics the prediction scenario of newly developed lines (predicting untested genotypes in observed environments). Models and terms are described in detail in the *Materials and Methods* section (Eqs 1–4). Horizontal and vertical dashed lines indicate the 20, 50, and 80% empirical percentiles corresponding to the observed and predicted values; the numbers inside the grid provide the conditional proportions observed on the *y*-axis for the different percentiles on the *x*-axis (e.g., out of the top 20% of the predicted values with M3 (panel C), 48% [top right] of these correspond to phenotypes that showed a performance among the 20% in fields).

**FIGURE 3:**
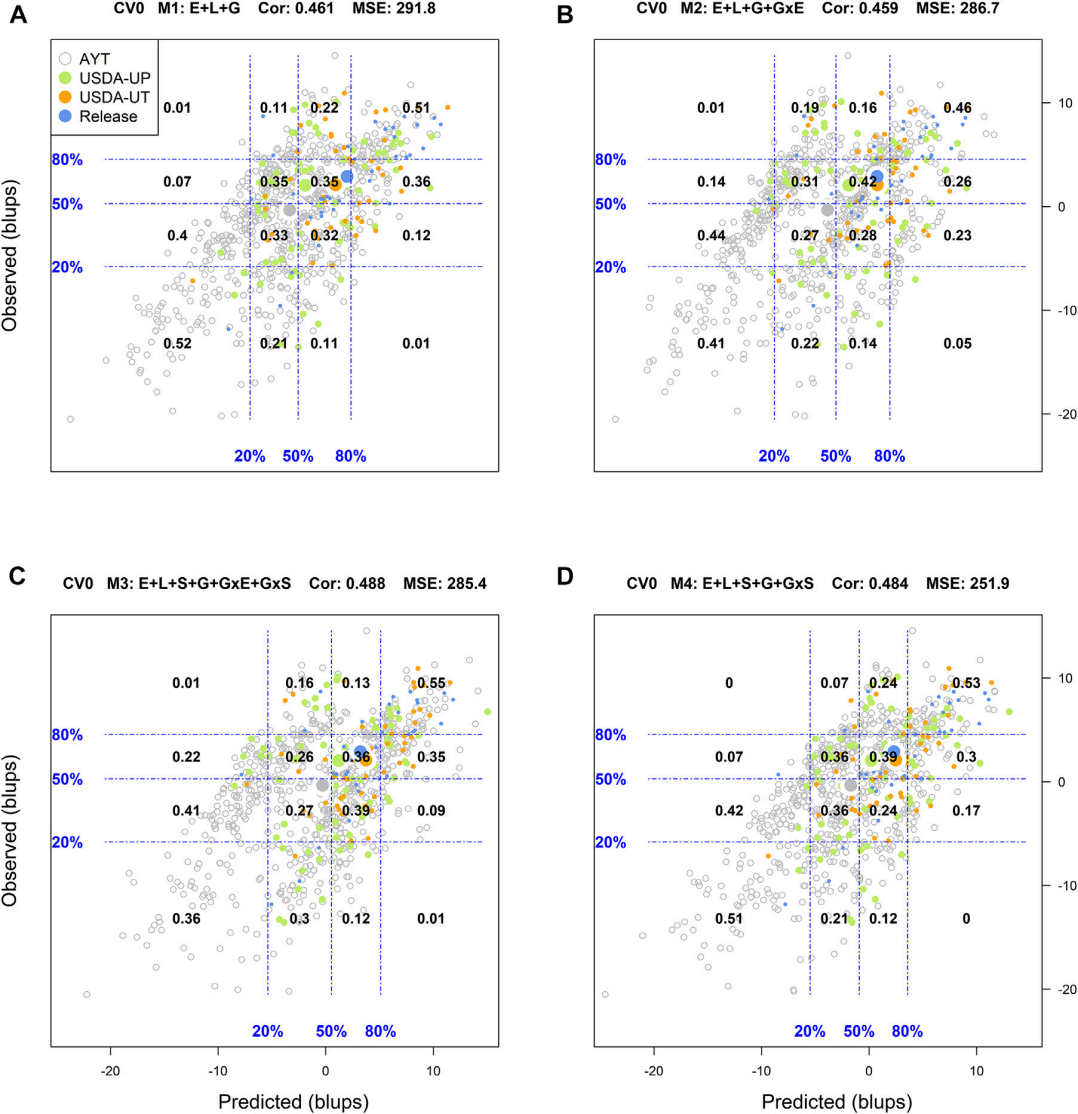
Genotypic means (BLUP-centered on zero within environments) of observed versus predicted cross-validation predictions of four models (M1–M4) under the cross-validation scheme CV0, which mimics the prediction scenario of predicting in novel environments (predicting tested genotypes in unobserved environments). Models and terms are described in detail in the *Materials and Methods* section (Eqs 1–4). Horizontal and vertical dashed lines indicate the 20, 50, and 80% empirical percentiles corresponding to the observed and predicted values; the numbers inside the grid provide the conditional proportions observed on the *y*-axis for the different percentiles in the *x*-axis (e.g., out of the top 20% of the predicted values with M3 (panel C), 55% [top right] of these correspond to phenotypes that showed a performance among the 20% in fields).

**FIGURE 4:**
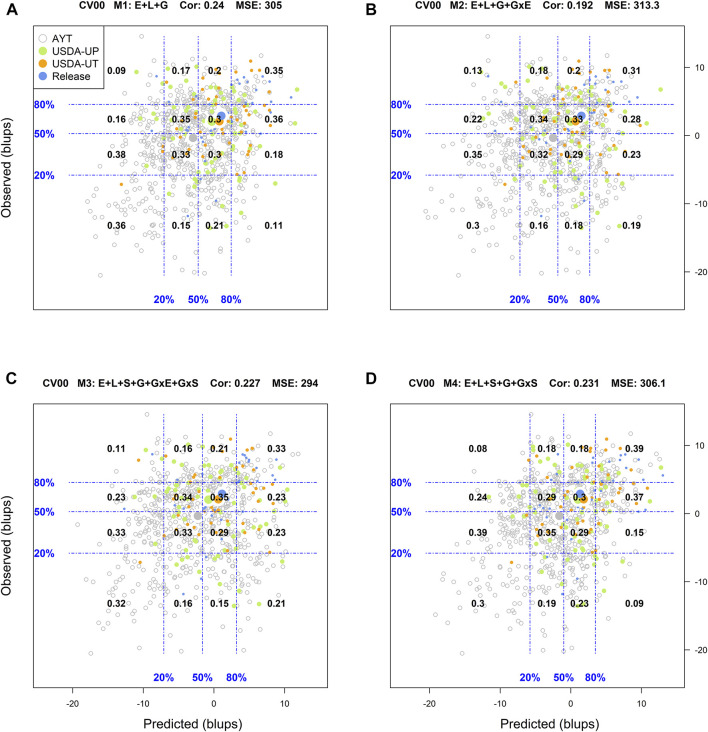
Genotypic means (BLUP-centered on zero within environments) of observed versus predicted cross-validation predictions of four models (M1–M4) under the cross-validation scheme CV00, which mimics the prediction scenario of predicting newly developed lines in novel environments (predicting untested genotypes in unobserved environments). Models and terms are described in detail in the *Materials and Methods* section (Eqs 1–4). Horizontal and vertical dashed lines indicate the 20, 50, and 80% empirical percentiles corresponding to the observed and predicted values; the numbers inside the grid provide the conditional proportions observed on the *y*-axis for the different percentiles on the *x*-axis (e.g., out of the top 20% of the predicted values with M4 (panel D), 39% [top right] of these correspond to phenotypes that showed a performance among the 20% in fields).

Another approach used to assess the model performance was the overall performance of the genotypes. For this, within each environment, the phenotypic and predicted values of all genotypes were adjusted by their corresponding environmental mean (centered on zero) followed by the computation of the across environment mean for all lines. Figures 1–4 display the classification success of the adjusted genotypes (observed and predicted values) marked by the advancement fate of each genotype including the advanced yield trial (AYT, gray), USDA Preliminary trials (USDA-UP, yellow), USDA Uniform trials (USDA-UT, orange), and Commercial Release (Release, blue). Detailed information on each stage of the breeding pipeline and selection criteria for line advancement were reported in Vieira and Chen (2021).

Figure 1 displays the results corresponding to the CV2 scenario. M1 returned a classification success of 76% for the top 20% of the predicted (adjusted) genotypes, and 79% success for the bottom 20% of the genotypes. In addition, the means of the predictions corresponding to the different advancement fate aligns with their counterpart based on phenotypes. There, the mean of the adjusted genotypes of the release (blue) group was superior followed by USDA-UT (orange), USDA-UP (yellow), and AYT (gray). Regarding M2 and M3, improvements in the classification accuracy were observed as compared to M1. With M3, a classification success of 76% was obtained for those genotypes in the top 20 and 79% for those in the lowest 20%. M4 returned intermediate results between M1 and M3 (Figure 1).

Similar to CV2, the corresponding results of CV1 are displayed in Figure 2. As expected, predicting new genotypes resulted in a significant reduction of the predictive ability of the models. With M1, the classification success of the top and bottom 20% of the predicted genotypes was 0.45, and 0.48, respectively. In this cross-validation scheme, the best results were shown in M2 with a classification success of 48% of the genotypes in the top 20 and 52% in the bottom 20%. Model M3 was the second-best model with the corresponding values for top and bottom 20% of 48 and 51%, respectively.

Regarding the prediction of the overall performance of tested genotypes in untested environments (CV0), M1 returned a classification success of 51 and 52% for the top and bottom 20%, respectively. The best results predicting the top 20% of the genotypes were obtained with M3 (55%), while M1 was the best (52%) for the bottom 20%. Model 4 produced intermediated results and it was the most stable across the diagonal in the grid (i.e., including the other percentiles), whereas M2 returned the poorest performance.

Finally, for the most complex prediction scenario CV00, M1 returned a classification success of 35% for the top 20 and 36% for the bottom 20%. M1 was the most accurate model in classifying genotypes with the poorest performance. M4 outperformed this model in the identification of the superior genotypes with a success rate of 39%. The remaining models underperformed M1 in identifying genotypes in both extremes, where M3 was slightly superior to M4 in the bottom 20% (0.32 vs. 0.30).

## Discussion

As the fields of genomics and data analytics substantially evolved over the past decade, the concept of genomic selection applied to phenotypic prediction revolutionized commercial and public breeding programs by allowing plant breeders to predict the phenotype of interest in untested genotypes (Crossa et al., 2017; Vieira and Chen, 2021; Wartha and Lorenz, 2021). Genomic selection has covered multiple fronts of the breeder’s equation maximizing the genetic gain in a given breeding cycle. For instance, a large component of a breeding cycle is allocated to progeny selection and preliminary yield trials of which the main objective is to characterize the genetic diversity present in a population of interest by evaluating a large number of genotypes for yield and overall agronomic traits. Genomic selection rises as a statistically powerful solution generating predicted values for unobserved genotypes, allowing plant breeders to shorten the breeding cycle and significantly minimize the costs associated with extensive field trials (Vieira and Chen, 2021; Wartha and Lorenz, 2021). Up to this date, however, the wide and large-scale implementation of genomic selection across plant breeding programs still faces challenges and drawbacks.

It is well-known that the expression of a phenotype is a function of the genotype, the environment, and the interaction between the genotype and environment (G×E) providing the relative performance of genotypes across different environments (Kang, 1997; de Leon et al., 2016). The differential response of genotypes across environments for a given phenotype of interest guide critical decisions in a plant breeding program, including the selection and advancement of genotypes as well as overall logistics and allocation of resources for multi-environment trials (Hill, 1975; Cooper and DeLacy, 1994; Kang, 1997; de Leon et al., 2016). Yield is a highly complex and quantitative trait regulated by numerous large and small-effect genes, of which its expression is immensely dependable on the genotype interaction with various components of the environment including pathogens (Rincker et al., 2017; Vieira et al., 2021), pests (Haile et al., 1998; Rocha et al., 2015), weeds (Oerke, 2006; Soltani et al., 2017), temperature, light, and precipitation (Runge and Odell, 1960; Goldblum, 2009; Alsajri et al., 2020), and soil-derived factors (Cox et al., 2003; Kaspar et al., 2004; Anthony et al., 2012). Thus, a practical and accurate implementation of genomic selection for yield relies on understanding and accounting for the interaction of molecular markers with the environment and/or its multiple components.

In this research, we aimed to expand the reaction norm model initially proposed by Jarquin et al. (2014b) which accounts for the interaction between molecular markers and the environment through covariance structures. Here, we investigated the potential of incorporating soil-derived covariates to enhance the predictive ability of yield across multiple cross-validation scenarios simulating progeny testing and line selection. A straightforward approach to examine the relative contribution of each model term is through the computation of variance components. Across environments, we observed that the addition of the G×S interaction in M3 substantially decreased the amount of variability captured by both the environment (−30.4%) and residual (−39.2%) terms as compared to the conventional GBLUP model (M1). When compared to the reaction norm model (M2), the addition of G×S equally reduced the amount of variability captured by the environment (−30.4%). Within environments, a larger reduction in variability captured by the residual term was observed in M3. Interestingly, the addition of the G×S term in M3 reduced the variability captured by the residual term by roughly 60 and 30% when compared to M1 and M2, respectively. The addition of soil-derived covariates seems to structure/dissect the environment term revealing components of the environment that could potentially enhance or hinder the performance of a model. This creates opportunities to explore more complex and readily available environmental components, which through covariance structures, could allow the borrowing of information across environmental components enhancing the predictive ability in challenging cross-validation scenarios. For instance, the amount of variability explained by both G×E (20.0%) and G×S (17.5%) in M3 shows that the inclusion of these terms increases the proportion of variance accounted for by the model, and therefore, it can enhance its predictive ability.

In regards to the predictive ability of each model across the proposed cross-validation scenarios, M3 outperformed the other models in CV2, CV1, and CV0. The conventional genomic selection model (GBLUP, M1) was the best in CV00. In the incomplete field trial scenario (CV2), M3 substantially outperformed M1 (25.1%). The ability of the covariance structures to borrow information from already observed genotypes in tested environments increased the model’s performance. In this case, the addition of G×S provides a slight edge over the reaction norm model (M2, 4%), highlighting the benefit of accounting for possible interactions between markers and soil types in overall prediction accuracy. An alternative methodology to assess the practical accuracy of the model consisting of empirical percentiles of the predicted and observed values was proposed by Jarquin et al. (2014b). Here, we observed that with M3 the classification accuracy for the top and bottom 20% percentile was 0.79 and 0.81, respectively. This represents approximately a 4% increment in classification accuracy as compared to M1. All four models flawlessly avoided misclassifying a top 20% percentile genotype as a bottom 20% percentile and *vice versa*, encouraging the practical applications of genomic prediction for line selection throughout the breeding pipeline. These results provide an opportunity to reconsider the experimental design in field trials, including the number of replications as well as overall resource allocation in multi-environment field trials. The prediction models can precisely discard inferior genotypes with nearly full confidence reducing the need for extensive preliminary field trials.

In CV1, M2 and M3 performed approximately 34% better than M1. In this cross-validation scenario, the genotypes are untested but the environment has been already observed with a different set of genotypes. The covariance structures allow the borrowing of information from previously observed genotypes, especially the main effects of molecular markers and the interaction between the markers and the environment. However, the structuring of the environment through the addition of G×S did not yield any advantages in prediction accuracy as compared to M2. Jarquin et al. (2021) observed similar results in CV1 when including the interactions using only weather data. This was attributed to G×E sufficiently capturing the similarities among pairs of environments leaving limited variance left to be explained by G×S. In the cross-validation scenario aiming to predict the yield of already tested genotypes in unobserved environments (CV0), M3 outperformed M1 and M2 by roughly 6%. These results provide an opportunity to explore alternative multi-environment testing and resource allocation throughout line selection in a breeding program. For instance, by leveraging the information of molecular markers of a different set of observed genotypes and known environments, plant breeders may be able to simulate multiple yield trials in a given growing season substantially increasing statistical power and confidence in line selection and advancement without necessarily increasing the investment in field operations. Similarly, the results from CV0 support both the reduction in the number of physical locations and the simulation of yield trials across diverse untested environments. This can substantially reduce the overall cost of a breeding pipeline while simultaneously enhancing statistical power and confidence in identifying genotypes with superior yield and overall adaptability.

In the most challenging cross-validation scenario considering untested genotypes in unobserved environments (CV00), M3 substantially outperformed M2 (19%), whereas M4 slightly outperformed M3 by 2%. Here, it highlighted the main advantage of incorporating G×S and S in the model. As previously discussed, the soil texture is generally constant across years and readily available before the growing season whereas the environment is often unfeasible to be accurately predicted prior to the growing season. Therefore, the borrowing of information from both soil covariate and molecular markers (in interaction) resulted in higher prediction accuracies as compared to M2. Although M1 yielded the highest prediction accuracy among the four models, M4 showed superior classification accuracy in the top 20% empirical percentiles (12% advantage over M1). By considering the advancement fate of the genotypes included in this analysis (AYT, USDA-UP, USDA-UT, and Release), nearly all the genotypes commercially released are concentrated in the top 20% and 50% observed and predicted empirical percentiles. This shows that, although the model may misclassify the empirical percentiles and/or show relatively low prediction accuracy, it does not negatively affect the identification and selection of the very best genotypes that will eventually be commercially released. These results support the modernization of a conventional breeding pipeline by precisely eliminating inferior genotypes prior to any field testing. Nearly 2 years of a conventional breeding pipeline is devoted to the assessment of the entire pool of genotypes representing a breeding cycle (Vieira and Chen, 2021). After reaching desired homozygosity (F_4:5_), a large number of genotypes are tested in progeny rows to visually evaluate their yield potential and overall agronomic traits. Selected genotypes, often consisting of many inferior genotypes mistakenly selected by subjective standards, are then tested in preliminary multi-environment yield trials. As seemed in CV00, the implementation of genomic selection has the potential for eliminating 2 years of extensive field testing by predicting the breeding values of untested genotypes. Thus, the wide implementation of genomic selection throughout a breeding pipeline holds promising improvements in cost efficiency, shortening the duration of the cycle, and overall genetic gain.

## Conclusion

The increasing availability of high-dimensional genomic data has allowed breeding programs to implement genomic selection to optimize the efficiency of a given breeding pipeline. Although widely adopted in commercial programs, the application of genomic selection in the public sector still faces limitations associated with costs, data availability, and technical support. In this research, we investigated the potential of incorporating soil texture and its interaction with molecular markers through covariance structures to increase prediction accuracy. As an approach to structuring the environmental term, the inclusion of G×S was shown to benefit the predictive ability of the models across multiple cross-validation scenarios. It is hypothesized that the availability of the soil texture prior to the growing season may have been essential to maximizing the functionality of covariance structures, particularly in scenarios with untested genotypes in untested environments. In addition, we demonstrated the applications of genomic selection across multiple stages of a breeding pipeline through four different cross-validation scenarios. In both progeny testing and line selection, we highlight the potential of genomic selection to optimize the efficiency of a soybean breeding program and discuss the opportunities to reconsider field experimental designs, allocation of resources, and reduction of preliminary field trials. Further studies considering covariates that are readily available before the growing season are encouraged to better understand the effect of the environment and enhance predictive ability. In addition, alternative metrics to assess the true potential and applicability of a model should be investigated to embolden the wide implementation of genomic selection in the public sector.

## Data Availability

The datasets presented in this study can be found in online repositories. The name of the repository and link to the data can be found as follows: Dryad; https://doi.org/10.5061/dryad.z8w9ghxf9.
