# Supplementary material for: Incorporation of Soil-Derived Covariates in Progeny Testing and Line Selection to Enhance Genomic Prediction Accuracy in Soybean Breeding
Source: Front Genet. 2022 Sep 8;13:905824. doi: 10.3389/fgene.2022.905824 (PMC9493273; doi:10.3389/fgene.2022.905824)
Supplement: Supplementary file 1 [file Presentation-1.zip › Supplementary Material/Figure S1.pptx]

## Slide 1
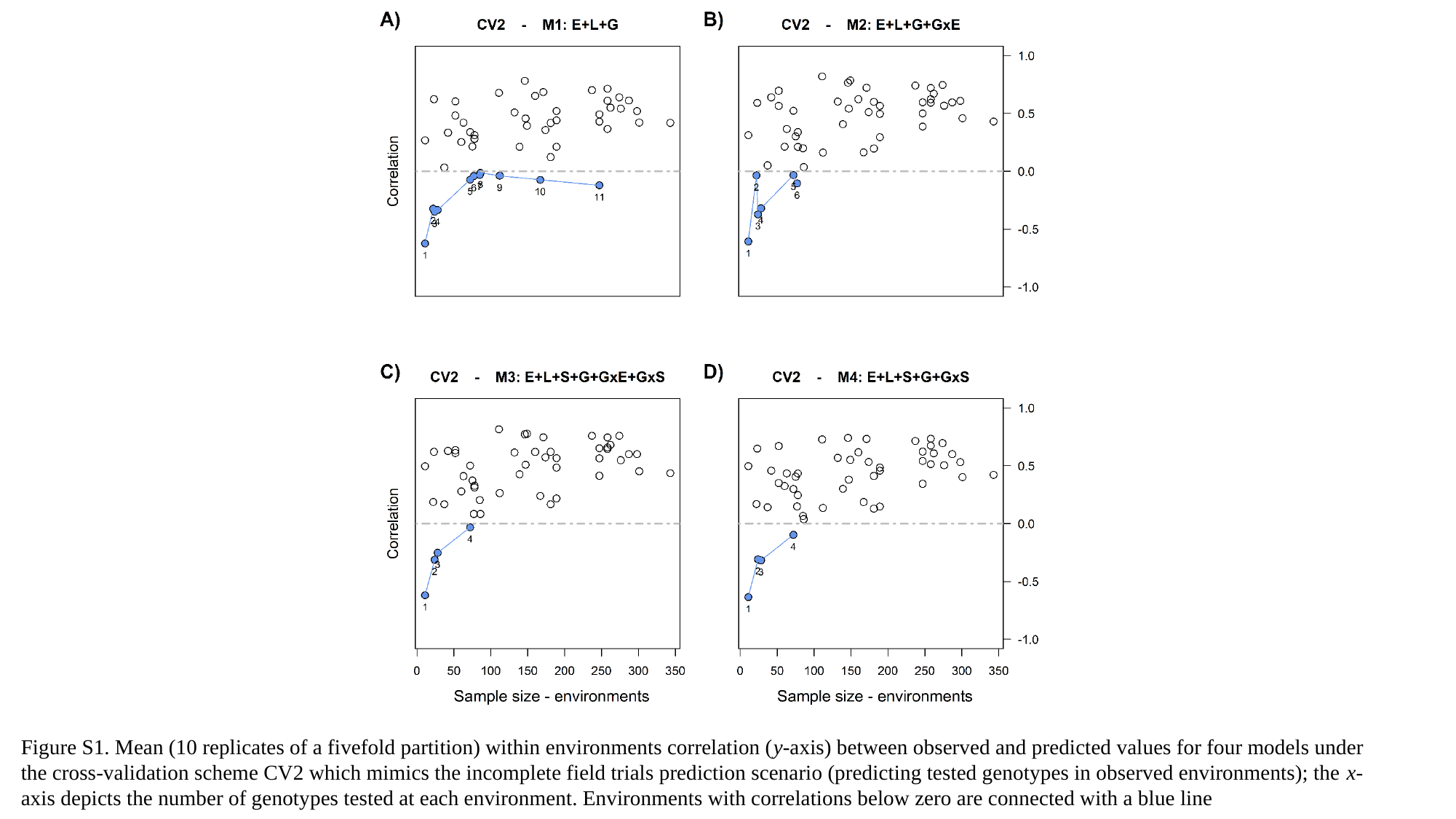

Figure S1. Mean (10 replicates of a fivefold partition) within environments correlation (y-axis) between observed and predicted values for four models under the cross-validation scheme CV2 which mimics the incomplete field trials prediction scenario (predicting tested genotypes in observed environments); the x-axis depicts the number of genotypes tested at each environment. Environments with correlations below zero are connected with a blue line
