# Supplementary material for: Incorporation of Soil-Derived Covariates in Progeny Testing and Line Selection to Enhance Genomic Prediction Accuracy in Soybean Breeding
Source: Front Genet. 2022 Sep 8;13:905824. doi: 10.3389/fgene.2022.905824 (PMC9493273; doi:10.3389/fgene.2022.905824)
Supplement: Supplementary file 1 [file Presentation-1.zip › Supplementary Material/Figure S6.pptx]

## Slide 1
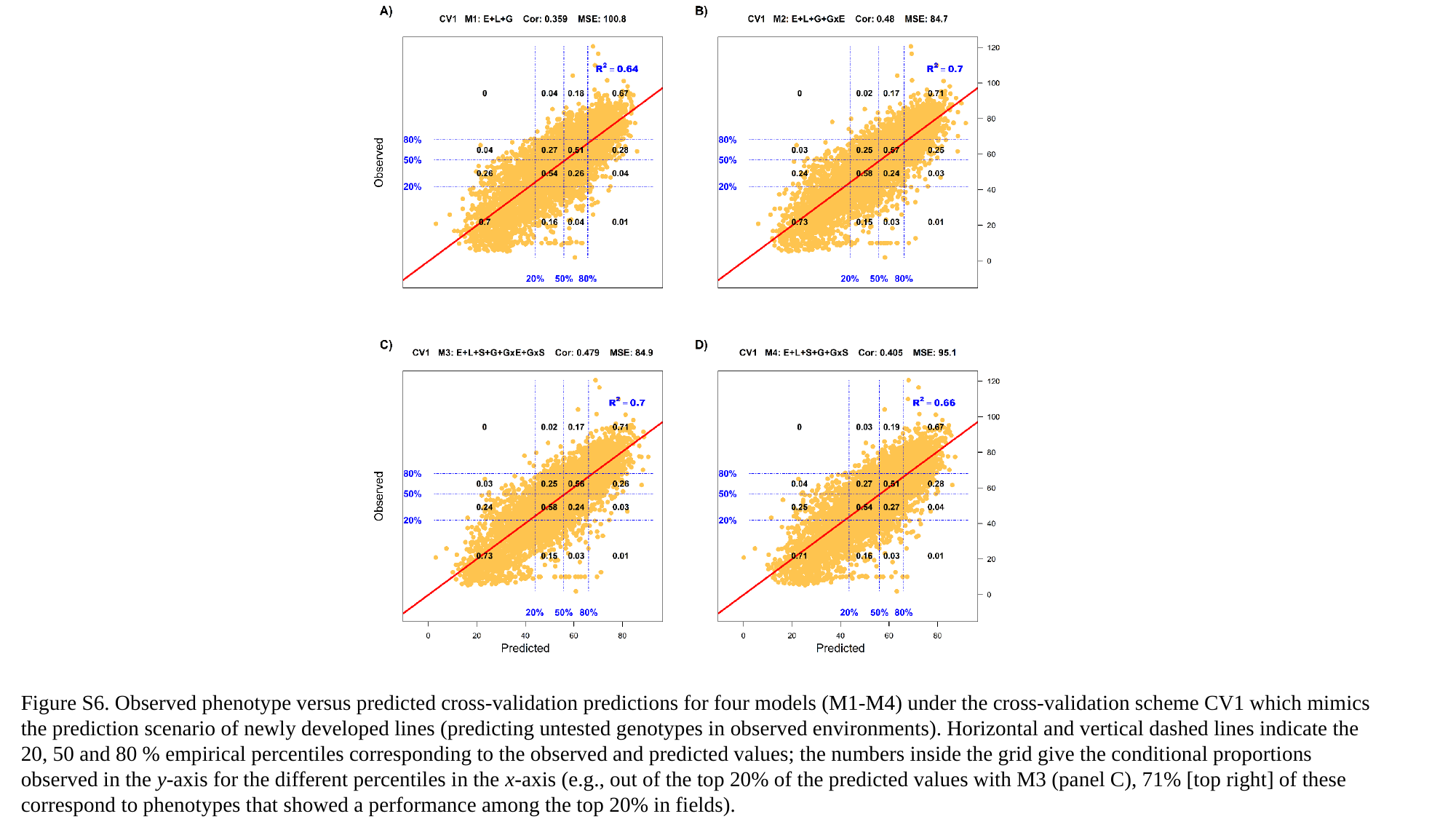

Figure S6. Observed phenotype versus predicted cross-validation predictions for four models (M1-M4) under the cross-validation scheme CV1 which mimics the prediction scenario of newly developed lines (predicting untested genotypes in observed environments). Horizontal and vertical dashed lines indicate the 20, 50 and 80 % empirical percentiles corresponding to the observed and predicted values; the numbers inside the grid give the conditional proportions observed in the y-axis for the different percentiles in the x-axis (e.g., out of the top 20% of the predicted values with M3 (panel C), 71% [top right] of these correspond to phenotypes that showed a performance among the top 20% in fields).
