# Supplementary material for: Incorporation of Soil-Derived Covariates in Progeny Testing and Line Selection to Enhance Genomic Prediction Accuracy in Soybean Breeding
Source: Front Genet. 2022 Sep 8;13:905824. doi: 10.3389/fgene.2022.905824 (PMC9493273; doi:10.3389/fgene.2022.905824)
Supplement: Supplementary file 1 [file Presentation-1.zip › Supplementary Material/Figure S8.pptx]

## Slide 1
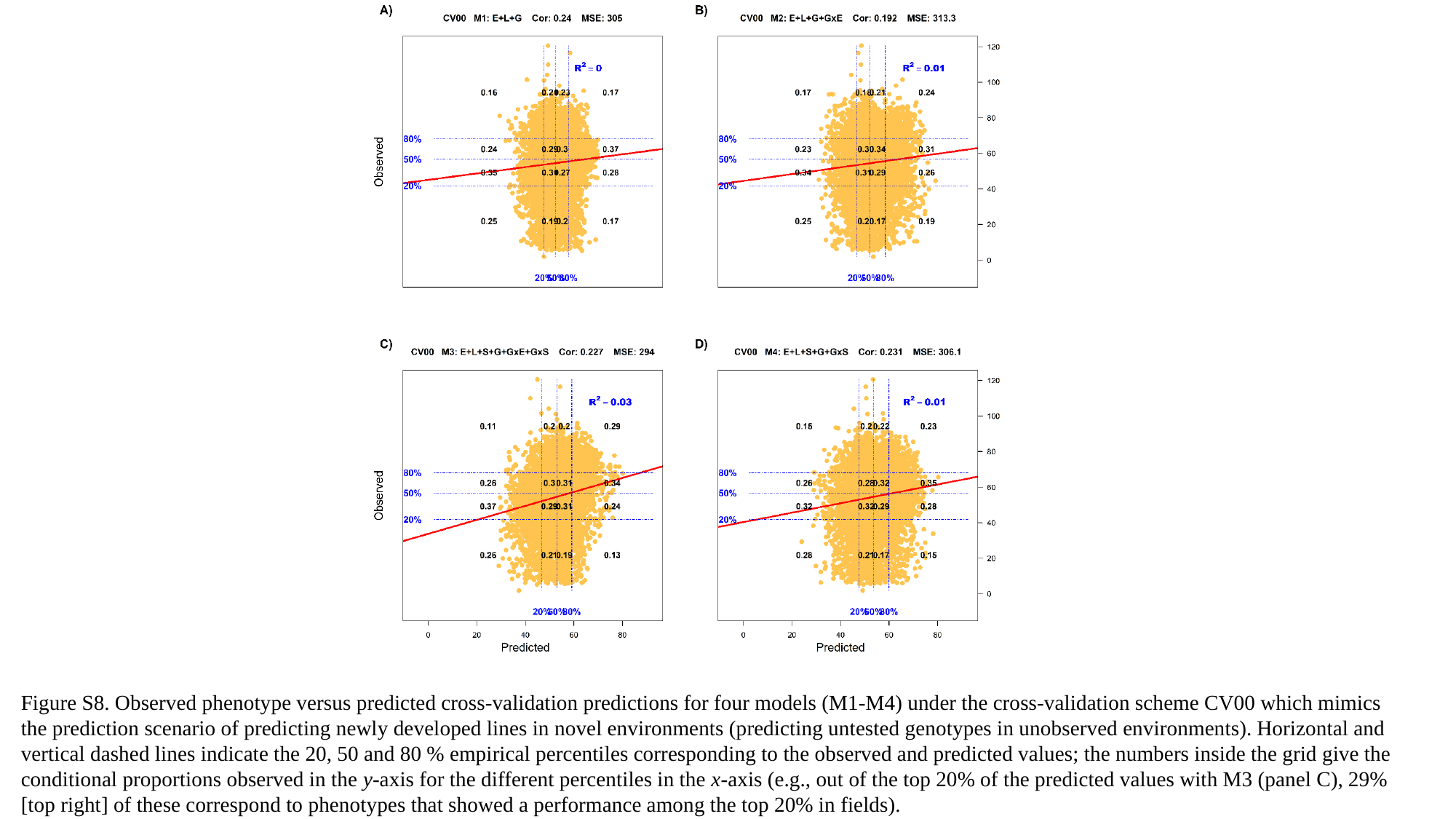

Figure S8. Observed phenotype versus predicted cross-validation predictions for four models (M1-M4) under the cross-validation scheme CV00 which mimics the prediction scenario of predicting newly developed lines in novel environments (predicting untested genotypes in unobserved environments). Horizontal and vertical dashed lines indicate the 20, 50 and 80 % empirical percentiles corresponding to the observed and predicted values; the numbers inside the grid give the conditional proportions observed in the y-axis for the different percentiles in the x-axis (e.g., out of the top 20% of the predicted values with M3 (panel C), 29% [top right] of these correspond to phenotypes that showed a performance among the top 20% in fields).
