# Supplementary material for: Incorporation of Soil-Derived Covariates in Progeny Testing and Line Selection to Enhance Genomic Prediction Accuracy in Soybean Breeding
Source: Front Genet. 2022 Sep 8;13:905824. doi: 10.3389/fgene.2022.905824 (PMC9493273; doi:10.3389/fgene.2022.905824)
Supplement: Supplementary file 1 [file Presentation-1.zip › Supplementary Material/Table S1.docx]

Table S1. Mean (10 replicates of a fivefold partition) and standard deviation (SD) of the within environments correlation between observed and predicted values for four models under the cross-validation scheme CV2 which mimics the incomplete field trials prediction scenario (predicting tested genotypes in observed environments).

| **Environment** | **Sample Size** | **M1:  E+L+G** | |  | **M2:  E+L+G+G×E** | |  | **M3: E+L+S+G+G×E+G×S** | |  | **M4:  E+L+S+G+G×S** | |
| --- | --- | --- | --- | --- | --- | --- | --- | --- | --- | --- | --- | --- |
|  |  | **Mean** | **SD** |  | **Mean** | **SD** |  | **Mean** | **SD** |  | **Mean** | **SD** |
| 2017_FLD_12_4 | 52 | 0.589 | 0.030 |  | 0.548 | 0.034 |  | 0.615 | 0.033 |  | 0.653 | 0.031 |
| 2017_FLD_12_5 | 42 | 0.322 | 0.040 |  | 0.623 | 0.034 |  | 0.615 | 0.037 |  | 0.446 | 0.031 |
| 2017_FLD_5_1 | 149 | 0.387 | 0.015 |  | 0.777 | 0.009 |  | 0.768 | 0.010 |  | 0.540 | 0.018 |
| 2017_FLD_5_2 | 37 | 0.033 | 0.048 |  | 0.049 | 0.087 |  | 0.149 | 0.091 |  | 0.130 | 0.063 |
| 2017_FLD_5_3 | 52 | 0.464 | 0.028 |  | 0.680 | 0.020 |  | 0.597 | 0.028 |  | 0.340 | 0.041 |
| 2017_FLD_6_1 | 28 | -0.323 | 0.067 |  | -0.300 | 0.093 |  | -0.233 | 0.083 |  | -0.298 | 0.060 |
| 2017_FLD_6_2 | 160 | 0.642 | 0.017 |  | 0.609 | 0.022 |  | 0.605 | 0.020 |  | 0.603 | 0.019 |
| 2017_FLD_8_1 | 63 | 0.412 | 0.020 |  | 0.354 | 0.034 |  | 0.394 | 0.028 |  | 0.424 | 0.016 |
| 2017_FLD_8_2 | 171 | 0.675 | 0.013 |  | 0.711 | 0.009 |  | 0.735 | 0.011 |  | 0.720 | 0.013 |
| 2017_FLD_8_7 | 146 | 0.767 | 0.011 |  | 0.754 | 0.016 |  | 0.760 | 0.017 |  | 0.728 | 0.012 |
| 2017_Rng_7 | 139 | 0.207 | 0.018 |  | 0.392 | 0.024 |  | 0.407 | 0.027 |  | 0.288 | 0.024 |
| 2018_FLD_12_5 | 189 | 0.208 | 0.021 |  | 0.281 | 0.029 |  | 0.208 | 0.024 |  | 0.145 | 0.014 |
| 2018_FLD_5_1 | 72 | -0.070 | 0.017 |  | -0.030 | 0.040 |  | -0.029 | 0.026 |  | -0.092 | 0.021 |
| 2018_FLD_5_2 | 181 | 0.410 | 0.013 |  | 0.586 | 0.022 |  | 0.604 | 0.022 |  | 0.398 | 0.027 |
| 2018_FLD_6_1 | 189 | 0.432 | 0.014 |  | 0.477 | 0.022 |  | 0.465 | 0.022 |  | 0.469 | 0.023 |
| 2018_FLD_6_2 | 85 | -0.031 | 0.019 |  | 0.188 | 0.050 |  | 0.194 | 0.055 |  | 0.062 | 0.035 |
| 2018_FLD_8_1 | 181 | 0.120 | 0.017 |  | 0.187 | 0.024 |  | 0.160 | 0.024 |  | 0.127 | 0.017 |
| 2018_FLD_8_2 | 86 | -0.013 | 0.021 |  | 0.034 | 0.031 |  | 0.080 | 0.022 |  | 0.037 | 0.021 |
| 2018_FLD_8_6 | 72 | 0.325 | 0.029 |  | 0.501 | 0.038 |  | 0.483 | 0.029 |  | 0.290 | 0.021 |
| 2018_Rng_10 | 132 | 0.497 | 0.014 |  | 0.590 | 0.019 |  | 0.599 | 0.023 |  | 0.546 | 0.028 |
| 2019_FLD_10_3 | 189 | 0.514 | 0.012 |  | 0.550 | 0.025 |  | 0.548 | 0.019 |  | 0.448 | 0.013 |
| 2019_FLD_12_5 | 23 | 0.616 | 0.032 |  | 0.583 | 0.028 |  | 0.613 | 0.033 |  | 0.642 | 0.033 |
| 2019_FLD_5_1 | 276 | 0.533 | 0.012 |  | 0.551 | 0.017 |  | 0.531 | 0.018 |  | 0.492 | 0.015 |
| 2019_FLD_5_2 | 24 | -0.339 | 0.064 |  | -0.346 | 0.121 |  | -0.293 | 0.110 |  | -0.294 | 0.066 |
| 2019_FLD_6_3 | 301 | 0.415 | 0.009 |  | 0.445 | 0.006 |  | 0.438 | 0.008 |  | 0.392 | 0.005 |
| 2019_FLD_8_2 | 274 | 0.627 | 0.012 |  | 0.737 | 0.009 |  | 0.749 | 0.008 |  | 0.683 | 0.008 |
| 2019_FLD_8_7 | 258 | 0.701 | 0.008 |  | 0.711 | 0.008 |  | 0.734 | 0.008 |  | 0.724 | 0.009 |
| 2019_FLD_8_8 | 237 | 0.690 | 0.011 |  | 0.730 | 0.009 |  | 0.748 | 0.009 |  | 0.704 | 0.008 |
| 2019_Rng_5 | 111 | 0.662 | 0.014 |  | 0.812 | 0.015 |  | 0.805 | 0.015 |  | 0.703 | 0.022 |
| 2019_Rng_6 | 167 | -0.072 | 0.019 |  | 0.151 | 0.038 |  | 0.222 | 0.032 |  | 0.178 | 0.023 |
| 2019_Rng_7 | 112 | -0.038 | 0.020 |  | 0.152 | 0.043 |  | 0.248 | 0.037 |  | 0.132 | 0.018 |
| 2020_FLD_12_5 | 75 | 0.201 | 0.049 |  | 0.276 | 0.059 |  | 0.350 | 0.045 |  | 0.386 | 0.032 |
| 2020_FLD_14_3 | 343 | 0.411 | 0.011 |  | 0.416 | 0.023 |  | 0.423 | 0.024 |  | 0.415 | 0.018 |
| 2020_FLD_14_4 | 77 | -0.038 | 0.065 |  | -0.089 | 0.051 |  | 0.074 | 0.052 |  | 0.141 | 0.047 |
| 2020_FLD_5_3 | 11 | 0.248 | 0.155 |  | 0.295 | 0.101 |  | 0.458 | 0.140 |  | 0.452 | 0.136 |
| 2020_FLD_6_1 | 287 | 0.603 | 0.011 |  | 0.575 | 0.019 |  | 0.581 | 0.018 |  | 0.589 | 0.010 |
| 2020_FLD_6_2 | 147 | 0.444 | 0.029 |  | 0.521 | 0.024 |  | 0.492 | 0.024 |  | 0.371 | 0.022 |
| 2020_FLD_6_3 | 262 | 0.539 | 0.011 |  | 0.651 | 0.022 |  | 0.659 | 0.023 |  | 0.588 | 0.023 |
| 2020_FLD_6_4 | 78 | 0.264 | 0.047 |  | 0.198 | 0.042 |  | 0.308 | 0.041 |  | 0.409 | 0.041 |
| 2020_FLD_6_5 | 78 | 0.294 | 0.029 |  | 0.320 | 0.043 |  | 0.295 | 0.045 |  | 0.234 | 0.031 |
| 2020_FLD_8_1 | 298 | 0.512 | 0.017 |  | 0.589 | 0.027 |  | 0.583 | 0.027 |  | 0.522 | 0.018 |
| 2020_FLD_8_6 | 60 | 0.241 | 0.044 |  | 0.198 | 0.065 |  | 0.260 | 0.052 |  | 0.311 | 0.035 |
| 2020_FLD_9 | 11 | -0.585 | 0.094 |  | -0.549 | 0.093 |  | -0.571 | 0.101 |  | -0.601 | 0.084 |
| 2020_Rng_8 | 22 | -0.267 | 0.092 |  | -0.030 | 0.145 |  | 0.172 | 0.107 |  | 0.154 | 0.079 |
| 2020_Rng_9 | 174 | 0.352 | 0.012 |  | 0.497 | 0.014 |  | 0.554 | 0.012 |  | 0.513 | 0.017 |
| 2021_FLD_6_3 | 258 | 0.358 | 0.018 |  | 0.609 | 0.016 |  | 0.630 | 0.014 |  | 0.501 | 0.018 |
| 2021_FLD_6_5 | 247 | 0.419 | 0.010 |  | 0.484 | 0.015 |  | 0.546 | 0.015 |  | 0.527 | 0.009 |
| 2021_FLD_8_1 | 258 | 0.594 | 0.016 |  | 0.573 | 0.018 |  | 0.637 | 0.018 |  | 0.655 | 0.018 |
| 2021_FLD_8_2 | 247 | 0.479 | 0.017 |  | 0.576 | 0.020 |  | 0.630 | 0.023 |  | 0.604 | 0.020 |
| 2021_FLD_CK | 247 | -0.118 | 0.015 |  | 0.371 | 0.031 |  | 0.397 | 0.026 |  | 0.332 | 0.021 |
