# Supplementary material for: Incorporation of Soil-Derived Covariates in Progeny Testing and Line Selection to Enhance Genomic Prediction Accuracy in Soybean Breeding
Source: Front Genet. 2022 Sep 8;13:905824. doi: 10.3389/fgene.2022.905824 (PMC9493273; doi:10.3389/fgene.2022.905824)
Supplement: Supplementary file 1 [file Presentation-1.zip › Supplementary Material/Table S2.docx]

Table S2 Mean (10 replicates of a fivefold partition) and standard deviation (SD) of the within environments correlation between observed and predicted values for four models under the cross-validation scheme CV1 which mimics the prediction scenario of newly developed genotypes in environments where other genotypes were already observed (predicting untested genotypes in observed environments).

| **Environment** | **Sample Size** | **M1:  E+L+G** | | **M2:  E+L+G+G×E** | | **M3:  E+L+S+G+G×E+G×S** | | **M4:  E+L+S+G+G×S** | |
| --- | --- | --- | --- | --- | --- | --- | --- | --- | --- |
|  |  | **Mean** | **SD** | **Mean** | **SD** | **Mean** | **SD** | **Mean** | **SD** |
| 2017_FLD_12_4 | 52 | 0.472 | 0.027 | 0.460 | 0.034 | 0.481 | 0.034 | 0.524 | 0.024 |
| 2017_FLD_12_5 | 42 | 0.418 | 0.051 | 0.633 | 0.058 | 0.574 | 0.063 | 0.435 | 0.054 |
| 2017_FLD_5_1 | 149 | 0.341 | 0.017 | 0.752 | 0.011 | 0.740 | 0.013 | 0.524 | 0.019 |
| 2017_FLD_5_2 | 37 | 0.069 | 0.053 | 0.045 | 0.080 | 0.052 | 0.098 | 0.080 | 0.067 |
| 2017_FLD_5_3 | 52 | 0.359 | 0.052 | 0.635 | 0.032 | 0.585 | 0.040 | 0.339 | 0.059 |
| 2017_FLD_6_1 | 28 | -0.318 | 0.048 | -0.309 | 0.077 | -0.152 | 0.091 | -0.151 | 0.080 |
| 2017_FLD_6_2 | 160 | 0.432 | 0.022 | 0.434 | 0.029 | 0.440 | 0.027 | 0.421 | 0.020 |
| 2017_FLD_8_1 | 63 | 0.256 | 0.043 | 0.199 | 0.042 | 0.222 | 0.038 | 0.262 | 0.049 |
| 2017_FLD_8_2 | 171 | 0.589 | 0.013 | 0.620 | 0.020 | 0.624 | 0.016 | 0.608 | 0.011 |
| 2017_FLD_8_7 | 146 | 0.571 | 0.026 | 0.618 | 0.027 | 0.624 | 0.023 | 0.548 | 0.024 |
| 2017_Rng_7 | 139 | 0.068 | 0.033 | 0.283 | 0.029 | 0.290 | 0.041 | 0.170 | 0.030 |
| 2018_FLD_12_5 | 189 | 0.107 | 0.010 | 0.175 | 0.021 | 0.090 | 0.023 | 0.047 | 0.014 |
| 2018_FLD_5_1 | 72 | -0.106 | 0.032 | -0.039 | 0.045 | -0.059 | 0.054 | -0.143 | 0.042 |
| 2018_FLD_5_2 | 181 | 0.334 | 0.025 | 0.527 | 0.028 | 0.526 | 0.028 | 0.299 | 0.026 |
| 2018_FLD_6_1 | 189 | 0.312 | 0.010 | 0.370 | 0.019 | 0.351 | 0.019 | 0.337 | 0.012 |
| 2018_FLD_6_2 | 85 | -0.179 | 0.038 | 0.056 | 0.057 | 0.052 | 0.060 | -0.112 | 0.046 |
| 2018_FLD_8_1 | 181 | 0.092 | 0.015 | 0.174 | 0.029 | 0.111 | 0.026 | 0.078 | 0.015 |
| 2018_FLD_8_2 | 86 | 0.149 | 0.023 | 0.187 | 0.050 | 0.188 | 0.048 | 0.134 | 0.024 |
| 2018_FLD_8_6 | 72 | 0.234 | 0.041 | 0.420 | 0.042 | 0.355 | 0.052 | 0.166 | 0.048 |
| 2018_Rng_10 | 132 | 0.388 | 0.019 | 0.531 | 0.035 | 0.543 | 0.033 | 0.495 | 0.020 |
| 2019_FLD_10_3 | 189 | 0.410 | 0.021 | 0.464 | 0.028 | 0.406 | 0.024 | 0.293 | 0.021 |
| 2019_FLD_12_5 | 23 | 0.538 | 0.031 | 0.507 | 0.034 | 0.489 | 0.040 | 0.542 | 0.036 |
| 2019_FLD_5_1 | 276 | 0.410 | 0.012 | 0.431 | 0.027 | 0.422 | 0.026 | 0.376 | 0.013 |
| 2019_FLD_5_2 | 24 | -0.239 | 0.057 | -0.064 | 0.146 | 0.022 | 0.132 | -0.159 | 0.070 |
| 2019_FLD_6_3 | 301 | 0.340 | 0.007 | 0.370 | 0.018 | 0.364 | 0.018 | 0.314 | 0.011 |
| 2019_FLD_8_2 | 274 | 0.560 | 0.015 | 0.689 | 0.017 | 0.691 | 0.016 | 0.616 | 0.013 |
| 2019_FLD_8_7 | 258 | 0.594 | 0.011 | 0.621 | 0.014 | 0.623 | 0.013 | 0.608 | 0.014 |
| 2019_FLD_8_8 | 237 | 0.574 | 0.016 | 0.652 | 0.011 | 0.661 | 0.012 | 0.605 | 0.013 |
| 2019_Rng_5 | 111 | 0.621 | 0.044 | 0.788 | 0.012 | 0.785 | 0.016 | 0.685 | 0.028 |
| 2019_Rng_6 | 167 | -0.028 | 0.014 | 0.213 | 0.033 | 0.250 | 0.034 | 0.193 | 0.022 |
| 2019_Rng_7 | 112 | -0.045 | 0.022 | 0.124 | 0.037 | 0.169 | 0.032 | 0.092 | 0.033 |
| 2020_FLD_12_5 | 75 | 0.111 | 0.061 | 0.246 | 0.063 | 0.268 | 0.042 | 0.248 | 0.027 |
| 2020_FLD_14_3 | 343 | 0.386 | 0.011 | 0.396 | 0.024 | 0.401 | 0.023 | 0.394 | 0.013 |
| 2020_FLD_14_4 | 77 | 0.054 | 0.073 | -0.030 | 0.103 | 0.013 | 0.094 | 0.063 | 0.064 |
| 2020_FLD_5_3 | 11 | 0.339 | 0.060 | 0.460 | 0.044 | 0.400 | 0.073 | 0.332 | 0.091 |
| 2020_FLD_6_1 | 287 | 0.457 | 0.018 | 0.458 | 0.027 | 0.454 | 0.025 | 0.440 | 0.017 |
| 2020_FLD_6_2 | 147 | 0.386 | 0.034 | 0.465 | 0.042 | 0.448 | 0.045 | 0.345 | 0.034 |
| 2020_FLD_6_3 | 262 | 0.343 | 0.022 | 0.517 | 0.033 | 0.502 | 0.032 | 0.371 | 0.023 |
| 2020_FLD_6_4 | 78 | 0.271 | 0.051 | 0.212 | 0.049 | 0.227 | 0.048 | 0.276 | 0.044 |
| 2020_FLD_6_5 | 78 | 0.217 | 0.046 | 0.302 | 0.059 | 0.273 | 0.057 | 0.184 | 0.037 |
| 2020_FLD_8_1 | 298 | 0.391 | 0.019 | 0.494 | 0.025 | 0.492 | 0.025 | 0.420 | 0.016 |
| 2020_FLD_8_6 | 60 | 0.183 | 0.053 | 0.111 | 0.064 | 0.159 | 0.060 | 0.252 | 0.051 |
| 2020_FLD_9 | 11 | -0.669 | 0.072 | -0.689 | 0.086 | -0.712 | 0.069 | -0.696 | 0.052 |
| 2020_Rng_8 | 22 | 0.008 | 0.130 | 0.079 | 0.144 | 0.287 | 0.148 | 0.370 | 0.131 |
| 2020_Rng_9 | 174 | 0.318 | 0.018 | 0.478 | 0.030 | 0.495 | 0.022 | 0.458 | 0.028 |
| 2021_FLD_6_3 | 258 | 0.344 | 0.019 | 0.578 | 0.010 | 0.576 | 0.015 | 0.456 | 0.027 |
| 2021_FLD_6_5 | 247 | 0.354 | 0.015 | 0.433 | 0.026 | 0.441 | 0.026 | 0.409 | 0.018 |
| 2021_FLD_8_1 | 258 | 0.452 | 0.021 | 0.459 | 0.030 | 0.465 | 0.028 | 0.442 | 0.021 |
| 2021_FLD_8_2 | 247 | 0.348 | 0.020 | 0.458 | 0.016 | 0.461 | 0.015 | 0.411 | 0.018 |
| 2021_FLD_CK | 247 | -0.109 | 0.022 | 0.375 | 0.028 | 0.391 | 0.029 | 0.335 | 0.020 |
