# Supplementary material for: Incorporation of Soil-Derived Covariates in Progeny Testing and Line Selection to Enhance Genomic Prediction Accuracy in Soybean Breeding
Source: Front Genet. 2022 Sep 8;13:905824. doi: 10.3389/fgene.2022.905824 (PMC9493273; doi:10.3389/fgene.2022.905824)
Supplement: Supplementary file 1 [file Presentation-1.zip › Supplementary Material/Table S3.docx]

Table S3. Within environments correlation between observed and predicted values for four models under the cross-validation scheme CV0 which mimics the prediction scenario of observed genotypes in novel environments (predicting tested genotypes in unobserved environments).

| **Environment** | **Sample Size** | **M1: E+L+G** | **M2: E+L+G+G×E** | **M3: E+L+S+G+G×E+G×S** | **M4: E+L+S+G+G×S** |
| --- | --- | --- | --- | --- | --- |
| 2017_FLD_12_4 | 52 | 0.626 | 0.623 | 0.682 | 0.692 |
| 2017_FLD_12_5 | 42 | 0.376 | 0.326 | 0.466 | 0.475 |
| 2017_FLD_5_1 | 149 | 0.368 | 0.417 | 0.517 | 0.465 |
| 2017_FLD_5_2 | 37 | 0.093 | 0.134 | 0.337 | 0.253 |
| 2017_FLD_5_3 | 52 | 0.478 | 0.509 | 0.371 | 0.330 |
| 2017_FLD_6_1 | 28 | -0.286 | -0.283 | -0.211 | -0.272 |
| 2017_FLD_6_2 | 160 | 0.662 | 0.678 | 0.635 | 0.609 |
| 2017_FLD_8_1 | 63 | 0.433 | 0.445 | 0.431 | 0.433 |
| 2017_FLD_8_2 | 171 | 0.690 | 0.670 | 0.730 | 0.734 |
| 2017_FLD_8_7 | 146 | 0.781 | 0.791 | 0.739 | 0.716 |
| 2017_Rng_7 | 139 | 0.228 | 0.244 | 0.279 | 0.265 |
| 2018_FLD_12_5 | 189 | 0.229 | 0.260 | 0.182 | 0.162 |
| 2018_FLD_5_1 | 72 | -0.037 | -0.044 | -0.058 | -0.056 |
| 2018_FLD_5_2 | 181 | 0.421 | 0.414 | 0.359 | 0.383 |
| 2018_FLD_6_1 | 189 | 0.448 | 0.438 | 0.439 | 0.485 |
| 2018_FLD_6_2 | 85 | 0.018 | 0.058 | 0.123 | 0.105 |
| 2018_FLD_8_1 | 181 | 0.135 | 0.144 | 0.133 | 0.138 |
| 2018_FLD_8_2 | 86 | -0.015 | -0.035 | 0.014 | 0.038 |
| 2018_FLD_8_6 | 72 | 0.356 | 0.378 | 0.303 | 0.288 |
| 2018_Rng_10 | 132 | 0.499 | 0.498 | 0.567 | 0.505 |
| 2019_FLD_10_3 | 189 | 0.518 | 0.515 | 0.444 | 0.428 |
| 2019_FLD_12_5 | 23 | 0.642 | 0.648 | 0.689 | 0.658 |
| 2019_FLD_5_1 | 276 | 0.546 | 0.552 | 0.502 | 0.496 |
| 2019_FLD_5_2 | 24 | -0.317 | -0.322 | -0.236 | -0.264 |
| 2019_FLD_6_3 | 301 | 0.422 | 0.416 | 0.384 | 0.385 |
| 2019_FLD_8_2 | 274 | 0.606 | 0.597 | 0.639 | 0.637 |
| 2019_FLD_8_7 | 258 | 0.712 | 0.705 | 0.745 | 0.736 |
| 2019_FLD_8_8 | 237 | 0.694 | 0.692 | 0.719 | 0.687 |
| 2019_Rng_5 | 111 | 0.651 | 0.632 | 0.388 | 0.389 |
| 2019_Rng_6 | 167 | -0.066 | -0.079 | 0.060 | 0.083 |
| 2019_Rng_7 | 112 | -0.018 | -0.030 | 0.127 | 0.124 |
| 2020_FLD_12_5 | 75 | 0.203 | 0.239 | 0.391 | 0.410 |
| 2020_FLD_14_3 | 343 | 0.417 | 0.390 | 0.392 | 0.408 |
| 2020_FLD_14_4 | 77 | 0.017 | -0.012 | 0.186 | 0.199 |
| 2020_FLD_5_3 | 11 | 0.316 | 0.242 | 0.446 | 0.554 |
| 2020_FLD_6_1 | 287 | 0.619 | 0.629 | 0.607 | 0.596 |
| 2020_FLD_6_2 | 147 | 0.469 | 0.483 | 0.383 | 0.377 |
| 2020_FLD_6_3 | 262 | 0.539 | 0.549 | 0.580 | 0.577 |
| 2020_FLD_6_4 | 78 | 0.319 | 0.268 | 0.436 | 0.482 |
| 2020_FLD_6_5 | 78 | 0.324 | 0.248 | 0.211 | 0.231 |
| 2020_FLD_8_1 | 298 | 0.517 | 0.497 | 0.496 | 0.515 |
| 2020_FLD_8_6 | 60 | 0.276 | 0.246 | 0.333 | 0.348 |
| 2020_FLD_9 | 11 | -0.473 | -0.422 | -0.466 | -0.499 |
| 2020_Rng_8 | 22 | -0.126 | -0.242 | 0.274 | 0.247 |
| 2020_Rng_9 | 174 | 0.366 | 0.369 | 0.511 | 0.502 |
| 2021_FLD_6_3 | 258 | 0.326 | 0.321 | 0.427 | 0.441 |
| 2021_FLD_6_5 | 247 | 0.426 | 0.382 | 0.502 | 0.539 |
| 2021_FLD_8_1 | 258 | 0.613 | 0.612 | 0.669 | 0.674 |
| 2021_FLD_8_2 | 247 | 0.482 | 0.528 | 0.635 | 0.612 |
| 2021_FLD_CK | 247 | -0.162 | -0.166 | -0.047 | -0.014 |
