# Supplementary material for: Incorporation of Soil-Derived Covariates in Progeny Testing and Line Selection to Enhance Genomic Prediction Accuracy in Soybean Breeding
Source: Front Genet. 2022 Sep 8;13:905824. doi: 10.3389/fgene.2022.905824 (PMC9493273; doi:10.3389/fgene.2022.905824)
Supplement: Supplementary file 1 [file Presentation-1.zip › Supplementary Material/Table S4.docx]

Table S4. Within environments correlation between observed and predicted values for four models under the cross-validation scheme CV00 which mimics the prediction scenario of new genotypes in novel environments (predicting untested genotypes in unobserved environments).

| **Environment** | **Sample Size** | **M1: E+L+G** | **M2: E+L+G+G×E** | **M3: E+L+S+G+G×E+G×S** | **M4: E+L+S+G+G×S** |
| --- | --- | --- | --- | --- | --- |
| 2017_FLD_12_4 | 52 | 0.197 | 0.573 | 0.501 | 0.491 |
| 2017_FLD_12_5 | 42 | 0.431 | 0.388 | 0.310 | 0.434 |
| 2017_FLD_5_1 | 149 | 0.231 | 0.248 | 0.209 | 0.154 |
| 2017_FLD_5_2 | 37 | 0.050 | 0.101 | 0.098 | 0.106 |
| 2017_FLD_5_3 | 52 | 0.400 | 0.350 | 0.183 | 0.400 |
| 2017_FLD_6_1 | 28 | -0.295 | -0.113 | -0.150 | -0.141 |
| 2017_FLD_6_2 | 160 | 0.330 | 0.381 | 0.315 | 0.252 |
| 2017_FLD_8_1 | 63 | 0.272 | 0.199 | 0.245 | 0.251 |
| 2017_FLD_8_2 | 171 | 0.503 | 0.433 | 0.369 | 0.510 |
| 2017_FLD_8_7 | 146 | 0.404 | 0.216 | 0.411 | 0.399 |
| 2017_Rng_7 | 139 | 0.092 | -0.038 | -0.009 | 0.097 |
| 2018_FLD_12_5 | 189 | 0.015 | -0.002 | 0.102 | -0.042 |
| 2018_FLD_5_1 | 72 | -0.102 | -0.208 | -0.090 | -0.119 |
| 2018_FLD_5_2 | 181 | 0.290 | 0.167 | 0.142 | 0.111 |
| 2018_FLD_6_1 | 189 | 0.229 | 0.243 | 0.222 | 0.231 |
| 2018_FLD_6_2 | 85 | -0.125 | -0.077 | -0.015 | -0.158 |
| 2018_FLD_8_1 | 181 | 0.102 | 0.112 | 0.068 | 0.103 |
| 2018_FLD_8_2 | 86 | 0.234 | 0.001 | 0.239 | 0.246 |
| 2018_FLD_8_6 | 72 | 0.196 | 0.267 | 0.113 | 0.091 |
| 2018_Rng_10 | 132 | 0.345 | 0.277 | 0.345 | 0.354 |
| 2019_FLD_10_3 | 189 | 0.350 | 0.297 | 0.152 | 0.149 |
| 2019_FLD_12_5 | 23 | 0.561 | 0.540 | 0.541 | 0.478 |
| 2019_FLD_5_1 | 276 | 0.294 | 0.202 | 0.149 | 0.242 |
| 2019_FLD_5_2 | 24 | -0.128 | -0.130 | -0.197 | 0.155 |
| 2019_FLD_6_3 | 301 | 0.180 | 0.178 | 0.096 | 0.147 |
| 2019_FLD_8_2 | 274 | 0.351 | 0.349 | 0.350 | 0.416 |
| 2019_FLD_8_7 | 258 | 0.444 | 0.457 | 0.503 | 0.425 |
| 2019_FLD_8_8 | 237 | 0.436 | 0.201 | 0.330 | 0.346 |
| 2019_Rng_5 | 111 | 0.262 | 0.499 | 0.268 | 0.221 |
| 2019_Rng_6 | 167 | -0.078 | -0.102 | 0.070 | 0.048 |
| 2019_Rng_7 | 112 | -0.023 | -0.043 | 0.097 | 0.045 |
| 2020_FLD_12_5 | 75 | -0.033 | 0.022 | 0.419 | 0.080 |
| 2020_FLD_14_3 | 343 | 0.240 | 0.180 | 0.230 | 0.199 |
| 2020_FLD_14_4 | 77 | 0.060 | 0.172 | 0.125 | 0.168 |
| 2020_FLD_5_3 | 11 | 0.099 | 0.135 | 0.075 | 0.352 |
| 2020_FLD_6_1 | 287 | 0.282 | 0.284 | 0.358 | 0.223 |
| 2020_FLD_6_2 | 147 | 0.380 | 0.103 | 0.206 | 0.198 |
| 2020_FLD_6_3 | 262 | 0.292 | 0.119 | 0.186 | 0.178 |
| 2020_FLD_6_4 | 78 | 0.087 | 0.240 | 0.279 | 0.401 |
| 2020_FLD_6_5 | 78 | 0.162 | 0.124 | 0.154 | 0.197 |
| 2020_FLD_8_1 | 298 | 0.292 | 0.156 | 0.287 | 0.156 |
| 2020_FLD_8_6 | 60 | 0.159 | 0.137 | 0.184 | 0.250 |
| 2020_FLD_9 | 11 | -0.690 | -0.635 | -0.673 | -0.717 |
| 2020_Rng_8 | 22 | 0.224 | 0.278 | 0.240 | 0.060 |
| 2020_Rng_9 | 174 | 0.324 | 0.296 | 0.345 | 0.327 |
| 2021_FLD_6_3 | 258 | 0.204 | 0.116 | 0.098 | 0.317 |
| 2021_FLD_6_5 | 247 | 0.264 | 0.182 | 0.315 | 0.263 |
| 2021_FLD_8_1 | 258 | 0.275 | 0.194 | 0.270 | 0.342 |
| 2021_FLD_8_2 | 247 | 0.212 | 0.157 | 0.183 | 0.333 |
| 2021_FLD_CK | 247 | -0.096 | -0.111 | 0.032 | 0.043 |
